# Genome recoding by tRNA modifications

**DOI:** 10.1098/rsob.160287

**Published:** 2016-12-14

**Authors:** Francesca Tuorto, Frank Lyko

**Affiliations:** Division of Epigenetics, DKFZ-ZMBH Alliance, German Cancer Research Center, Heidelberg, Germany

**Keywords:** tRNA modification, tRNA anticodon, protein translation, genome recoding

## Abstract

RNA modifications are emerging as an additional regulatory layer on top of the primary RNA sequence. These modifications are particularly enriched in tRNAs where they can regulate not only global protein translation, but also protein translation at the codon level. Modifications located in or in the vicinity of tRNA anticodons are highly conserved in eukaryotes and have been identified as potential regulators of mRNA decoding. Recent studies have provided novel insights into how these modifications orchestrate the speed and fidelity of translation to ensure proper protein homeostasis. This review highlights the prominent modifications in the tRNA anticodon loop: queuosine, inosine, 5-methoxycarbonylmethyl-2-thiouridine, wybutosine, threonyl–carbamoyl–adenosine and 5-methylcytosine. We discuss the functional relevance of these modifications in protein translation and their emerging role in eukaryotic genome recoding during cellular adaptation and disease.

## 1. Introduction

All ribonucleic acid (RNA) species carry modified nucleosides that have been implicated in various biological roles, such as RNA homeostasis, coding, decoding, regulation and expression of genes [1,2]. RNA modifications are particularly enriched in tRNAs, with over 80 modifications reported [3–5]. Many modifications within the structural core of the tRNA are essential for stabilizing the overall molecular structure; loss of these modifications can result in rapid degradation of hypomodified tRNAs [6]. The most diverse and complex chemical structures are found in the anticodon stem loop, either in the anticodon at the wobble position or directly adjacent to it [7,8]. Loss of these modifications can reduce protein production or translational accuracy, suggesting that the chemical complexity is necessary to maintain optimum translational processivity.

The succession of mRNA codons controls the synthesis of polypeptides through the complementarity between each of the 64 possible codon triplets and the tRNA anticodons that decode the 20 amino acids of the cellular proteome. Central to the mRNA decoding process is the backward compatibility of the codon : anticodon recognition that is mediated by tRNA. The first and second base of the codon and the third and second base of the anticodon interact following the Watson–Crick pairing rules (A : U, U : A, G : C, C : G). In contrast, the interaction between the third base of the codon and the first base of the anticodon (position 34) is less constrained, as proposed by Francis Crick in his wobble hypothesis [9]. Crick also predicted the possibility of G : U wobbling and the pairing of I with U, C and A with a preference for the two pyrimidine bases. As a result, a given tRNA may read more than one synonymous codon. Indeed, 597 tRNA genes have been identified so far with 57 different anticodons decoding the standard 20 amino acids in the human genome [10].

On the basis of the wobble rules, a minimum of 32 anticodons is needed to decode the 61 sense codons in mRNAs. However, several genetic systems encode fewer tRNA genes than this minimal set. Examples include organelles (plastids and mitochondria) and some parasitic bacteria, such as mycoplasms. Two main mechanisms have been suggested to explain how translation occurs with a reduced tRNA set: two out of three decoding [11] and superwobble decoding [12]. Two out of three decoding postulates that a tRNA pairing with only the first two codon bases can be sufficient for translation and that any base can occur at the wobble codon position. This would apply for those codon families that have a high GC content and thus form strong GC base pairs with the two pairing nucleotides of the codon–anticodon interaction. The suggested alternative hypothesis (‘superwobble’ or ‘four-way wobble’) suggests that four nucleotides in a codon family can be decoded by a single tRNA species with an unmodified U in the wobble position [13].

Recent progress in identifying modified nucleotides and their functions in tRNA [1,14], and information gained from detailed structural, physico-chemical and kinetic studies of ribosomes associated with mRNA/aminoacyl-tRNA, have made clear that an integrative interaction network between mRNA, tRNA and rRNA ensures translation fidelity [15].

**Figure 1.**
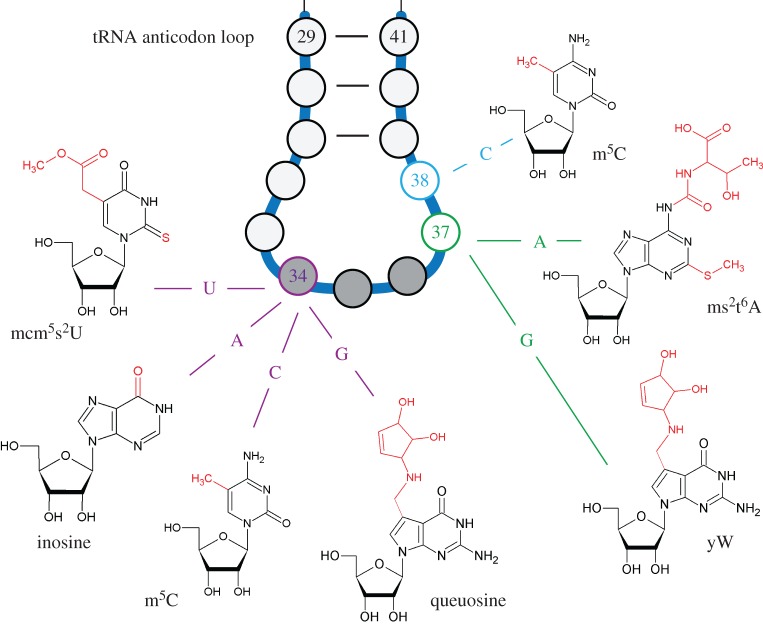
Selected modified ribonucleosides in the tRNA anticodon stem and loop of the eukaryotes. Positions 34 and 37 of the anticodon loop are subject to various post-transcriptional modifications. Highlighted are modified nucleosides ensuring correct decoding at the wobble position (34), and modifications at positions 37 and 38 that play roles in reading frame maintenance and fidelity.

tRNA modifications play a key role in the codon : anticodon pairing and decoding process [8]. The greatest diversity of hypermodified nucleotides occurs at positions 34 and 37 of the anticodon of tRNAs (figure 1). Modifications at these positions ensure base pairing flexibility during decoding and reading frame maintenance [3,16], and have been shown to expand the ability of tRNAs to read additional codons [8]. In particular, position 34, corresponding to the first base of the anticodon loop of tRNAs, is subject to various modifications, depending on the associated tRNA isoacceptor and the organism [5,17].

Eukaryotic tRNA modifications and enzymes have been extensively characterized in the yeast *Saccharomyces cerevisiae* [6,18,19]. Recent advances in next-generation sequencing and mass spectrometry have revealed the importance of those modifications also in higher eukaryotes [20]. tRNA modifications are generally present in the same locations derived from the specificity of the modification enzymes and tRNA structure. Accurate quantification of modified nucleosides at high sensitivity has emerged as an important challenge, as the modification patterns of tRNAs were found to vary as a function of various types of stress [21].

A large number of methods are based on separation of modified nucleotides. The physico-chemical properties of the single nucleosides permit their separation, and also serve for their identification and characterization by retention values. The combination of ^32^P-labelling and two-dimensional TLC separation on cellulose has been used to detect more than 70 modifications [22,23]. The presently most sensitive and accurate quantification methods rely on mass spectrometry, as LC–MS/MS allows the quantitative detection of modifications in the low femtomolar range [24–26]. Furthermore, the specific position of modified nucleosides in tRNAs can be now identified by combining the isolation of specific tRNAs with enzymatic digestion and LC–MS/MS [27,28]. Further information about the sequence context of modifications can be obtained by RNA bisulfite sequencing, which allows the detection of (cytosine 5) RNA methylation marks at single-base resolution [29]. This method holds substantial promise for the comprehensive characterization of transcriptome-wide RNA methylation patterns. Similarly, ARM-seq (AlkB-facilitated RNA methylation sequencing) or DM-tRNA-seq (demethylase tRNA sequencing) revealed a complex modification landscape of full-length tRNAs and tRNA fragments [30–32].

Finally, it is also important to investigate how nucleoside modifications influence the translational efficiency at the codon level. In this context, ribosome profiling is an emerging technique that uses next-generation sequencing to monitor *in vivo* translation and allows identification of the amount of specific proteins that are produced by cells [33]. As translating ribosomes produce footprints on the mRNA, the position of these footprints can be used to measure the time a ribosome spends on a particular codon. If a ribosome stalls at a specific codon, an increase of the respective footprint will be observed, and this information can be used to determine codon-specific translation elongation rates. Together, these technological advances provide novel insights into how tRNA modifications affect mRNA decoding.

In the following sections, we discuss five distinct modifications that are found at or in the vicinity of tRNA anticodons and that have been connected to the control of protein translation (figure 1). In the final section of this review, we develop a mechanistic framework for how these modifications can be used for translational genome recoding.

## 2. Queuosine

**Figure 2.**
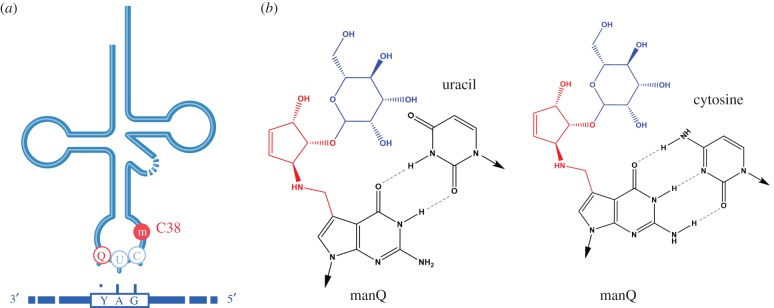
Effects of tRNA modifications on mRNA decoding. (*a*) Illustration of 5-methylcytidine at C38 and queuosine at G34 in the anticodon loop of tRNA-Asp and its relation to the codon of the mRNA. (*b*) Binding of mannosyl-queuosine to cytosine and uracil. Arrows point towards the primary ribose moiety, which is not shown.

Queuosine (Q) is a hypermodified nucleoside that occurs at the wobble position of tRNAs with GUN anticodons, where N represents any nucleotide (N = G, A, T, C). Interestingly, in eukaryotic organisms, only tRNA genes with GUN anticodons have been found to translate NAC/U codons. The translation of NAU is mediated by base modifications of the anticodon tRNA loop, which adapt its geometry to the mRNA codon in the ribosome (figure 2). These features have established Q as an early paradigm for the concept of tRNA modification-based genome recoding.

tRNA queuosinylation is mediated by the tRNA-guanine transglycosylase (TGT) complex. This complex consists of the catalytic subunit, Q-tRNA-ribosyltransferase 1 (Qtrt1), and a homologous accessory subunit, Q-tRNA-ribosyltransferase domain containing 1 (Qtrtd1). The complex incorporates queuine into cytosolic tRNA-Tyr, -Asn, -Asp and -His, and into mitochondrial tRNA-Asp [34]. tRNA-Asp and tRNA-Tyr are further modified to mannosyl Q-tRNA (manQ34) and galactosyl Q-tRNA (galQ34), respectively [35].

Even if Q is present in eukaryotic cells, only bacteria can synthesize Q de novo. Studies on germ-free (axenic) mice maintained on a chemically defined diet provided clear evidence that eukaryotes are non-autotrophic for queuosine biosynthesis [36]. More specifically, germ-free mice fed with a queuine-free diet were found to have reduced queuosine modification levels of tRNAs, and exogenous administration of queuine restored queuosine modification levels [36,37]. Animals obtain Q or its analogues as a micronutrient from dietary sources and from the gut microbiota. This establishes Q as a particularly interesting modification that links tRNA modification to ‘environmental’ variables. In animal cells, changes in the abundance of Q have been shown to correlate with diverse phenomena, including stress tolerance, cell proliferation and tumor growth. However, the difficulty of maintaining animals under bacteria-free conditions on Q-deficient diets has severely hampered the study of Q metabolism and its function in animals. As such, the molecular mechanisms underlying these phenotypes are not yet understood. Interestingly, however, recent data from *Drosophila* suggest that the presence of queuosine in tRNA alters translational fidelity [38]. This provides a key mechanism for the control of protein translation by the nutritional environment and the gut microbiome (see below).

## 3. Inosine

RNA editing is a post-transcriptional process in which a genomically templated sequence is altered at the RNA level. It is distinguished from other forms of RNA modification in that the consequence of RNA editing is a change that increases genetic diversity [39]. In tRNAs, the most common editing mechanism involves base deamination: ‘programmed changes’ of one canonical nucleotide for another that may impact tRNA overall structure and function [39]. The most prominent type of deamination involves the conversion of adenosine (A) to inosine (I), and has been observed in Archaea, Bacteria and Eukarya. Additionally, tRNAs may also undergo cytosine (C) to uridine (U) editing, which has been described in Archaea, marsupials, kinetoplastids and plant organelles [40,41]. Both A–I editing and C–U editing affect the anticodon, changing the decoding ability from one codon to another and effectively expanding the decoding properties of the edited tRNA [41].

In mammals, inosine is a post-transcriptional modification found at three different positions in tRNAs: position 34, 37 and 57. It is the result of a deamination reaction of adenines that is catalysed by adenosine deaminases acting on tRNAs (ADATs). The homodimeric enzyme ADAT1 generates inosine at position 37 only in eukaryotic tRNA-Ala, where it is also further modified into 1-methylinosine (m^1^I37). In yeast, knockouts of ADAT1 are viable, suggesting that m^1^I37 is not an essential tRNA modification [42]. Inosine 57 is only present in archaea as 1-methylinosine (m^1^I57), and both its function and the catalysing enzyme are currently unknown [43]. Inosine at position 34 (I34) expands the tRNA decoding capacity and it has been described for every ANN tRNA, whereas A34 can, in principle, only pair with codons having a U at the third codon position; I34 can pair with U-, C- and A-ended codons [9].

Editing is catalysed by the heterodimeric enzyme ADAT (hetADAT), which is composed of two subunits: ADAT2 and ADAT3 [44]. Eukaryotic I34 and ADAT have been characterized *in vivo* in yeast [45], *Trypanosoma brucei* [41] and recently in *Arabidopsis*, where the tRNA adenosine deaminase arginine (TADA) gene encodes a deaminase responsible for the editing of the adenosine at the wobble position of tRNA-Arg (ACG). A mutation in *TADA* leads to slower chloroplast translation, causing profound effects on chloroplast function and plant development [40].

Adenine-to-inosine editing of tRNA anticodons is used by both eukaryotes and prokaryotes to expand the decoding capacity of individual tRNAs and to limit the number of tRNA species required for codon–anticodon recognition [46]. Nevertheless, the phenotypic consequences of the lack of inosine editing on tRNAs in metazoans have only recently been addressed [47]. In humans, knockdown of ADAT2 modulates the levels of I34 editing on tRNA substrates of the heterodimeric ADAT complex. While these fluctuations are tolerated by the cells [47], I34 hypomodification has also been associated with myositis [48]. Furthermore, a missense mutation in the ADAT3 gene has been associated with intellectual disability and strabismus in eight different consanguineous families [49], thus indicating a functional relevance of the I34 editing for human health and disease [47].

## 4. U34 thiolation and related modifications

In eukaryotes, the U34 base of 11 cytoplasmic tRNAs carries a 5-methoxycarbonylmethyl (mcm^5^) or 5-carbamoylmethyl (ncm^5^) modification. The addition of these moieties requires the six-subunit Elongator (Elp) complex, a protein complex initially identified as a component of a hyperphosphorylated RNA polymerase II holoenzyme isolated from budding yeast chromatin [50]. Orthologues of Elp2–Elp4 are conserved in humans, with two additional proteins that are presumably analogous to Elp5 and Elp6 from yeast [51]. Initially identified as a transcriptional elongation complex in the nucleus, the Elp complex finally turned out to be a cytoplasmic complex that regulates translational efficiency by adding mcm5 and ncm5 groups on uridines at the wobble position [52]. While Elp3 was found to be the catalytic subunit of the Elongator protein complex [53], Elp3 expression is induced by Wnt signalling and is essential for Wnt-driven tumour development in the intestine [54]. Recently, Elp3 has also been linked to gene-specific translation during breast cancer progression [55]. Deletion of mouse Elp3 triggers ER stress and the unfolded protein response (UPR), thus impairing the generation of intermediate neuronal progenitors and leading to microcephaly [56].

Following mcm^5^U34 addition to three tRNAs (tRNA–GluUUC, tRNA–LysUUU, tRNA–GlnUUG), the U34 base is further modified with a 2-thio group resulting in a 5-methoxycarbonylmethyl-2-thiouridine (mcm^5^s^2^U) nucleotide via a sulfur-relay pathway that requires the ubiquitin ligase-like proteins Uba4, Urm1, Ncs2 and Ncs6. Many of the responsible modifying enzymes are conserved across eukaryotes [57]. Modification of U34 is generally considered to enhance the efficiency and fidelity of translation [16,58,59]. In a recent study in *S. cerevisiae* and *C. elegans*, lack of U34 modifications led to ribosome pausing at cognate codons. In addition, cells lacking U34 modifications exhibited gene expression hallmarks of proteotoxic stress, accumulated as aggregates, and were severely compromised in clearing stress-induced protein aggregates. Overexpression of hypomodified tRNAs alleviated ribosome pausing and concomitantly restored protein homeostasis [60]. These findings convincingly demonstrated a functional role of U34 thiolation for optimal codon translation and the maintenance of proteome integrity. Interestingly, studies in yeast have also linked tRNA thiolation to nutrient-dependent responses. It was shown that tRNA uridine thiolation abundance reflects sulfur-containing amino acid availability and functions to regulate translational capacity and amino acid homeostasis. Uridine thiolation therefore represents a key mechanism that coordinates protein translation and growth with metabolism [61].

## 5. Complex modifications at position 37

In most tRNAs, G37 is methylated at the base to form 1-methylguanosine (m^1^G), which is found in tRNAs in all domains of life [62]. In addition, 1-methylguanosine at position 37 of the anticodon loop (m^1^G37) also serves as a chemical platform for additional modifications, as shown for tRNA-Phe of Archaea and Eukarya. In these organisms, m^1^G is the first step in the formation of wybutosine (yW) in Eukarya, and wyosine (imG) and its derivatives in Archaea [63,64]. In *S. cerevisiae*, formation of yW requires five enzymes acting in a strictly sequential order: Trm5, Tyw1, Tyw2, Tyw3 and Tyw4 [63].

The presence of wyosine and its derivatives at position 37 of tRNA-Phe provides base-stacking interactions of the tRNA anticodon with the A-site codon that play a key function in reading frame maintenance by preventing the propensity for ribosome ‘slippage’ on the phenylalanine codons UUU and UUC [65]. Interestingly, this ‘frameshifting potential’ can also be used in a programmed manner to increase coding diversity. For example, many viruses rely on programmed +1 frameshifting to allow translation of multiple proteins or protein variants from a single promoter [66].

Additional anticodon loop hypermodifications can occur when position 37 is an adenosine. The tRNA isopentenyltransferases (IPTases) are conserved from bacteria to humans and introduce an evolutionarily ancient isopentenyl group onto N6 of adenine at position 37 (i^6^A37). Functional analysis in eukaryotes comes from studies in yeast, which have shown that i^6^A37 promotes translational efficiency and fidelity at cognate codons, but decreases fidelity at non-cognate codons [67].

The enzymes involved in t6A synthesis were only identified and characterized over the last few years [68]. In yeast, components of the threonyl–carbamoyl transferase complex (TCTC) are required for t^6^A synthesis. Tcs3p (Kae1p) and Tcs5p (Bud32p) are part of the TCTC complex. Mutation of either gene eliminates t^6^A in tRNA and causes pronounced slow-growth phenotypes [69]. In addition, modulation of t^6^A in *Drosophila* through expression of an unmodifiable tRNA-iMet or overexpression of *TCS3* led to alterations of Tor activity and changes in organismal growth. Additionally, knockdown of Tcs3 (Kae1) or Tcs5 (Bud32) in *Drosophila* larvae activated the UPR [70]. Analysis of codon occupancy rates by polysome profiling suggested that one of the major roles of t^6^A is to homogenize the process of elongation by slowing the elongation rate at codons that are decoded by high-abundance tRNAs and I34 : C3 pairs, while increasing the elongation rate of rare tRNAs and G34 : U3 pairs [71].

Lastly, CDKAL1 encodes a methylthiotransferase involved in the complex 2-methylthio-*N*6-threonyl carbamoyladenosine (ms^2^t^6^A) modification at position 37 in tRNA-LysUUU. Published evidence suggests that lack of ms^2^t^6^A37 leads to mistranslation of several proteins, including proinsulin. Abnormal proinsulin accumulates and cannot be converted into insulin, leading to glucose intolerance and type 2 diabetes [72]. These findings again support the notion that tRNA modifications involved in mRNA decoding are important for human health and disease.

## 6. C38 m^5^C

5-Methylcytosine is widely known in the context of DNA methylation and epigenetic gene regulation [73]. Interestingly, this modification has also been described in several cellular RNAs, and a complex enzymatic machinery for its synthesis was found in organisms from all kingdoms of life [74]. The recent development of RNA bisulfite sequencing puts m^5^C in a highly privileged position as one of the few RNA modifications that can be mapped at single-base resolution by sequencing methods [29].

In eukaryotic tRNAs, m^5^C residues are clustered at the junction between variable region and TΨC-stem, and positions 48 and 49 are the most frequently modified. In addition, higher eukaryotes frequently have an additional m^5^C residue in the tRNA acceptor stem, at position 72 [75]. C34 in the anticodon loop and C48, C49 and C50 in the TΨC extra loop are methylated by NSun2 [76,77], which belongs to the NSun-domain-containing family of RNA methyltransferases (NSun1–NSun7). Furthermore, NSun6 has been suggested to methylate C72 in the acceptor stem in tRNA-Cys and tRNA-Thr [78]. Besides the NSun enzymes, Dnmt2 currently represents the only other known cytosine-5 RNA methyltransferase in higher eukaryotes that targets tRNA substrates [79]. The analysis of double knockout Dnmt2/NSun2 mice and the deletion of *NSun2* in a tumour mouse model suggested a role of 5-methylcytosine in the regulation of global protein synthesis and cell fate [77,80]. Mechanistically, this was linked to the finding that 5-methylcytosine protects tRNAs against endonucleolytic cleavage [81,82], thus preserving the steady-state levels of substrate tRNAs and promoting global protein translation and differentiation [80].

Dnmt2 is a unique enzyme that uses the catalytic mechanism of eukaryotic DNA methyltransferases to methylate RNA [79]. In eukaryotes, the most conserved substrate for Dnmt2 is cytosine 38 (C38) of tRNA-Asp, which is a Dnmt2 target in a wide range of organisms, including protists, plants and animals [79]. For Dnmt2 mutants, a variety of phenotypes have been described, ranging from subtle, context-dependent changes to profound developmental defects [79,83]. However, the relationship between tRNA methylation and these phenotypes has remained unclear. We have recently performed a detailed analysis of the Dnmt2 mutant mouse phenotype, with a specific focus on the haematopoietic system as a paradigm for cellular proliferation and differentiation. Our analysis of phenotypically affected tissues from Dnmt2^−/−^ mice uncovered a novel function of Dnmt2 in the regulation of polypeptide synthesis [84]. Indeed, dysregulated proteins in Dnmt2^−/−^ bone marrow cells showed codon biases with increased rates of Asp amino acid mistranslation. Mass spectrometry analysis in combination with ribosome profiling suggested that, during translation of Asp codons, C38 tRNA methylation enables the discrimination of near-cognate codons and thereby contributes to the accuracy of polypeptide synthesis [84]. C38 methylation thus represents a modification that contributes to both tRNA stability and translational accuracy [77,84].

## 7. Genome recoding by tRNA modifications

mRNA sequences contain more information than the amino acid sequence and redundancy in the genetic code offers an opportunity for the fine-tuning of protein production [85]. Codon bias, which is defined by the frequencies of synonymous codons, is a specific characteristic of each genome and even each gene [86]. Phylogenetic analyses revealed that the codon usage bias and the tRNA gene content are adapted to each other [46,86]. Furthermore, the choice of synonymous codons can impact organism fitness, owing to the differences in the speed and accuracy with which they are read as a consequence of not only tRNA abundance, but also tRNA modifications. The overall modification level has been correlated with the *in vitro* protein synthesis capacity, suggesting that the extent to which the tRNA ensemble is chemically modified modulates the translational efficiency [25]. Moreover, anticodon modifications that expand the wobbling capacity increase the translation efficiency of the codons recognized by the modified tRNAs.

Although the biological impact of tRNA modifications on single-codon preference may be moderate, the cumulative effect across an entire genome could result in considerable changes. In this regard, it is interesting that a dramatic shift in codon preference has been identified across the drosophilid lineage with respect to the amino acids tyrosine, histidine, asparagine and aspartic acid, which are translated by tRNAs with GUN anticodons. These tRNAs share the queuosine modification at the wobble position. Moreover, *Drosophila melanogaster* showed a preference for NAC codons, whereas *Drosophila virilis* had a preference for NAU codons consistent with a shift in Q-tRNA modification between these two species [38]. Furthermore, Zaborske and co-workers [38] also observed an accuracy-driven selection shift with Q modification across organism development. This suggests a ‘kinetic competition model’, where the presence of Q34 leads to a more accurate translation of the C-ending codon as a result of increased Q–C binding affinity. Indeed, in the absence of Q34, a U-ending codon is more accurate than a C-ending codon, because the competition from the wrong tRNA is weaker. In this way, Q-modification acts to reverse the relative codon accuracy within a dual synonymous codon family [38]. Because Q-modification is limited by the availability of queuine, this micronutrient acts to influence translational fidelity and ultimately the evolutionary trajectory of the fly genome [38].

**Figure 3.**
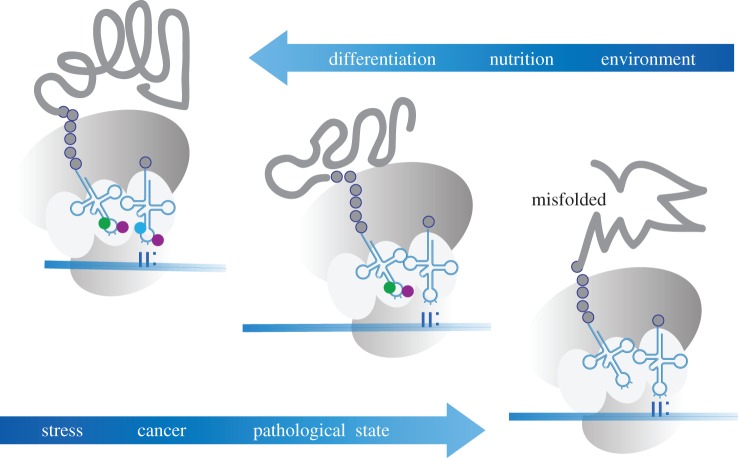
Modifications of the anticodon stem and loop regulate gene expression through altered decoding. Extrinsic factors that have been shown to promote these mechanisms include environmental conditions, nutritional states and cellular differentiation. The absence of modifications can result in misfolded proteins, and can be associated with stress, pathological states and cancer.

Interestingly, it has also been shown that C38 tRNA methylation in *Schizosaccharomyces pombe* is regulated by queuine [87]. As 5mC at C38 has been shown to have a function in translational accuracy [84], these results suggest a novel and exciting mechanism that allows nutritional factors to modulate mRNA decoding and translation. This possibility is also supported by a study describing a link between extracellular sulfur amino acids and U34 tRNA thiolation-dependent translational regulation of metabolic genes in yeast [61]. More specifically, tRNA uridine thiolation under amino-acid-rich conditions promoted the translation of mRNAs enriched in Lys, Gln and Glu residues, which are often found in genes that are involved in protein synthesis and growth control. However, when the sulfur amino acids methionine or cysteine were depleted, tRNA thiolation became reduced. This resulted in a decreased protein synthesis of growth factors and an increased expression of amino acid biosynthesis factors. As such, tRNA thiolation acts as a metabolic ‘switch’ in responses to amino acid availability, by controlling the translation of key metabolic genes [61]. The significance of U34 modifications in protein translation is further illustrated during the response to cellular stress. Here, it has been shown that the UPR can be modulated by tRNA U34 modifications through codon translation speed and that this mechanism contributes to the control of cortical neurogenesis during mammalian brain development [56]. These findings suggest that genome recoding by tRNA modifications represents an important mechanism for adapting cells and organisms to changing environmental conditions (figure 3) [88]. This hypothesis is also supported by observations that demonstrate highly dynamic changes of tRNA modifications under various stress conditions [21,89].

To comprehensively understand the effects of tRNA modifications on codon recognition and mRNA translation, it will also be necessary to consider the context provided by other modifications at the anticodon stem loop and their structural and biochemical impact [15]. Given that many organisms have adopted different decoding strategies based on alternative combinations of tRNA modifications within the same tRNA molecule [4], the relative contribution of a single tRNA modification to decoding might be species-specific. Particularly, in mammalian systems, many details remain to be understood, including the dynamics of tRNA modifications under physiological and pathological conditions. It is notable that enzymes catalysing tRNA modifications are often linked to human diseases, ranging from metabolic defects, mitochondrial dysfunctions and neurological disorders to cancer [90]. How tRNA modifications interact with the regulated control of translation and cell function is only beginning to be explored. Many tRNA modifications change during different phases of the cell cycle, suggesting that these modifications may also be relevant for cell cycle control and tumour growth [91,92]. It will be important to understand the dynamics and the molecular mechanisms that target tRNAs for modification and how these modifications affect protein translation in pathological states. Integrative high-throughput approaches including polysome profiling and mass spectrometry will undoubtedly provide us with novel opportunities for detailed insights into the corresponding mechanisms.
